# Preparation of medical personnel for an early response humanitarian mission – lessons learned from the Israeli defense forces field hospital in the Philippines

**DOI:** 10.1186/2054-314X-1-5

**Published:** 2015-02-24

**Authors:** Tomer Erlich, Avi Shina, David Segal, Tal Marom, David Dagan, Elon Glassberg

**Affiliations:** 3 Israel Defense Forces Medical Corps, Ramat Gan, Tel Hashomer, Israel; 4 Department of Urology, The Chaim Sheba Medical Center, Ramat Gan, Tel Hashomer, Israel; 5 Department of Obstetrics and Gynecology, The Chaim Sheba Medical Center, Ramat Gan, Tel Hashomer, Israel; 6 Department of Otolaryngology - Head & Neck Surgery, Edith Wolfson Medical Center, Tel Aviv University Sackler School of Medicine, Holon, Israel; 7 Trauma and Combat Medicine Branch, Surgeon General Headquarters, Medical Corps Israel Defense Forces, Ramat Gan, 02149 Israel

**Keywords:** Disaster, Humanitarian mission, Field hospital, Preparation, Check list

## Abstract

**Introduction:**

Humanitarian aid provision and early medical response missions to areas ravaged by natural disasters are as essential nowadays as in the past, and medical personnel play a pivotal role in these delegations.

**Case description:**

In November 2013, tropical cyclone Haiyan (Yolanda) slammed the Philippines archipelago, leaving more than an estimated 6000 dead in its wake while demolishing vital infrastructure and affecting the life of an estimated 25 million locals. The Israeli Defense Forces (IDF) rapidly constructed and sent a humanitarian aid delegation which included a field hospital deployment with medical capabilities from diverse specialty fields.

**Discussion and evaluation:**

The purpose of this article is to summarize our experience in the preparation process of medical personnel before and during deployment. We offer a simple, practical and structured checklist that will assist the medical specialist in preparation for his mission.

**Conclusion:**

Preparation of medical personnel for humanitarian aid medical missions is a complex and vital task that might be better accomplished with thorough briefing and structured checklists which begin with addressing of personal safety and other daily needs of the staff.

## Background

Unfortunately, natural disasters occur throughout history and at times cause mass casualties which warrant the rapid deployment of medical response aid missions to overcome the damage [1–3]. In November 2013, tropical cyclone *Haiyan* (Yolanda) slammed the Philippines archipelago, leaving more than an estimated 6000 dead in its wake while demolishing vital infrastructure and affecting the life of an estimated 25 million locals [4]. In the storms’ aftermath welfare infrastructures were severely damaged and capabilities of local healthcare services dramatically decreased. As in other massive natural disasters, the Israeli Government instructed the Israeli Defense Forces (IDF) to rapidly construct a humanitarian aid delegation which included a field hospital deployment with medical capabilities from diverse specialty fields [5]. The mission goal was to assist the local healthcare staff and facilities to overcome the tremendous damage and destruction to medical services and infrastructure.

Participating in a humanitarian aid mission is usually a gratifying and fulfilling endeavor but may also prove to be challenging task, full of uncertainties and difficulties especially when the delegation is rapidly assembled and deployed to an area hit with disaster of calamitous proportions. A simple but structured guideline may simplify the preparation phase for the team members. In this report, we sought to present our experience and lessons learned regarding the preparation of medical personnel for early response humanitarian aid delegations. We hope this will serve as a practical guideline and checklist for the team leader and medical specialist before embarking on such missions.

Our recommendations are based on our own and others’ personal experience, lessons learned from previous delegations deployed by the IDF Medical Corps, staff briefings, group sessions and after action reports filed upon returning to Israel.

## Case presentation

### Team composition

The IDF has gained experience in deploying early response medical and humanitarian aid delegations to disaster zones around the globe [1, 2] based on its ability to quickly recruit, prepare and equip medical and logistical personnel, some of which are called on through reserve duty. This medical aid delegation was assembled within hours and embarked to the Philippines. In cases of natural disasters with a multitude of casualties, rapid deployment is key in order to exert a beneficial effect to the local population in terms of morbidity and mortality [6]. As our delegation personnel was assembled from a relatively heterogeneous background, active and reserve duty alike, it required the appointed medical personnel to bring their everyday life to a sudden standstill and embark on an unknown and often disconcerting task [7].

Choosing the right personnel is one of the crucial components of successful mission planning and execution. We feel that putting together a delegation composed of health care providers with previous experience in natural disaster aid alongside first time participants is beneficial, as the more experienced members usually help alleviate stress with practical knowhow and thus ease the preparation process for all. Integration of younger less experienced members also ensures and maintenance of organizational memory in medical aid deployment. The tradeoff of this approach of course was an initial lack of cohesiveness but on the other hand, it enabled a refreshing and empowering experience for the novice participants. This form recruitment strategy also enabled a “tailor made” solution for the region we were to operate in as gathered from site survey feedback. It is prudent to recruit personnel known to withstand stressful environments if possible as this might reduce future repercussions [8]. Team leaders, mental health specialists (essential part of the delegation) should be notified of at risk individuals.

Thus, optimal personnel recruitment in the mission planning phase while still in the homeland is the initial and necessary step in achieving the mission’s goals [7].

### Briefing the team

Briefing the appointed personnel while in the homeland alleviates stress and clears uncertainties which may hamper personnel functionality. It is important to underscore the fact that the briefing will contain information mostly from what is known about pre-disaster conditions in the destination, as site survey feedback from the scout team is often partial, inaccurate and may be sent back too late to exert a real difference on the delegation. Flexibility must be stressed as a vital quality for the healthcare provider and the delegation as a whole.

We suggest that briefing should be given as soon as the task force is assembled and should include the following agenda:

## Destination’s geographic and sociological information

1. Destination area: world region, country, region, urban, rural, other.
2. Climate: day and night temperatures, humidity/rain/snow.
3. Local language: need for translators, basic words for medical communication.
4. Endemic diseases: need for additional vaccinations and prophylactic treatments.
5. Sanitary conditions: is there any flowing water? Is it potable?
6. Personal safety: crime, terrorism, precautions to be taken.
7. Religion and culture- with emphasis of customs related to practical medicine. For example: can a male physician examine a female? What are the mourning customs? What are the burial rites and customs? Is it acceptable to send tissue which was removed during surgery for a pathologic exam or must it be buried? Are milk substitutes or pacifiers acceptable for infants?

## Delegation structure and makeup

1. Mission goal- what are the main objectives as based on the needs of the local authorities, site survey feedback, personnel makeup and organizational orientation. Discuss team as well as personal expectations and set realistic goals for the delegation and the individual.
2. Delegation composition- who the personnel are, both medical, logistical and support staff. What is the appointed command chain?
3. Medical capabilities in surgical, intensive care (including pediatric), neonatal care, obstetrics, ancillary services (i.e. imaging, pharmacy and laboratory).
4. Basic organizational issues: routine schedule, sleeping and eating arrangements are important to emphasize. Delegation participants should be briefed on daily routines, as these tend to reduce uncertainty and create a sense of stability. Team leaders should display flexibility and urge delegation members to suggest necessary adjustments to the initial plan as the mission progresses.
5. Social support networks emphasis-Well-established social support networks were found to be beneficial in lowering the prevalence of mental implications. Recognition of this need, by both the individuals and the team leaders, is essential [7]. Providing the measures and encouraging delegation members to reach out to these networks back home (friends, family) or to bond and form new networks while in deployment should be emphasized as a part of the orientation and briefing.

Furthermore, as constant changes occur during the planning and execution of such missions, conceptual readiness for changes should be discussed as part of the briefing.

## Essential medical considerations

1. Level of care: in most medical delegations it will not be possible to provide the same level of medical care as practiced at the delegations’ origin country. Contingencies will have to be made and ethical dilemmas faced. Some of these dilemmas have been discussed elsewhere [9, 10] and it is beyond the scope of this article to address this aspect of the preparation process. Examples include the possible inability to conduct a pathologic examination for tissues removed during surgery in post-disaster conditions, decision making regarding incidental suspicious findings in the operating theatre and managing of surplus medical supplies at mission completion. Open staff discussion covering anticipated ethical dilemmas before deployment may assist the team upon arrival. It is essential to relay that ethical dilemmas are routine due to the extreme circumstances in natural disasters, have been encountered by the more experienced team members in past missions and should be resolved in open group discussions.
2. Work site: will an independent field hospital facility open or will the delegation work within or aside a local healthcare facility [5] and if so what will its capabilities include.
3. Collaboration with local or other healthcare personnel and facilities: the local population may receive care from a number of different caregivers and in these cases it is crucial to acknowledge the designated case manager and how continuity of care will be established upon mission completion.
4. Medical equipment: the appropriate type, quality and quantity of medical equipment are the keys for maximal humanitarian aid impact. Each specialist must personally verify his specific tools and medications. This should be done by a thorough review of a checklist composed beforehand while still in the homeland. Due to weight, space and other limitations, some equipment may be missing and the specialist may have to familiarize himself with several methods of performing medical procedures. It is not unusual that specialists may need to improvise at the scene if they lack their own tools or medications.
5. Medical routine: daily staff routine in the facility – timetable, shifts, rounds, staff meetings, patient admission and discharge procedures, ordering lab exams and radiology.
6. Patient case records: will the patient chart be recorded manually or digitally, in which language, and will he be given a hard copy. Will local physicians write the notes? Will records be left to the local staff?
7. Common practice issues:
8. Obtaining patient informed consent before procedures.
9. Labor and delivery- treatment of women in the purperium, providing neonatal care, follow up and recommendations.
10. Multitasking – Each care giver will be asked to be responsible for tasks different from his specialty (both medical and auxiliary such as imaging etc.).

### Personal gear

Early response medical humanitarian aid is usually based on governmental agencies, non- governmental organizations (NGOs) or military organizations. Therefore, in many cases, the organization will supply its own personal gear elements for the healthcare provider. Nevertheless, one should check that all the critical and optional equipment is present, according the geographic and sociological information which has been gathered. Table 1 shows our core recommended list of personal gear.

**Table 1:** **Personal gear checklist for the healthcare provider in an early response medical humanitarian mission**

Item	Details
Sandals	For shower and off-duty time
Toiletries	1. Towels
	2. Soap, shampoo
	3. Hand sanitizer
	4. Shaving kit and scissors
	5. Toilet paper for the first 3 days
	6. Female hygienic products
Sleeping equipment	1. Sleeping bag (may need to be waterproof)
	2. Personal Tent
	3. Bed linen
	4. Inflatable mattress
	5. Pillow
Head light + batteries	At least 2 sets of batteries
Communication device (cell phone) + charger + international wall plug	pre-departure arrangements of an appropriate program with the mobile company are recommended
Camera	Can be part of the communication device
General supply	Pens, pencils, notebooks, marker (to mark your equipment),umbrella
Personal medical equipment	1. Stethoscope, surgical eye loops etc
	2. Personal prescription medication
	3. Contraception
	4. Copy of medical diploma
Specialized medical equipment	In case certain gear requested will not be supplied by the delegation
Watch	
Musical instrument	Optional

## Discussion and evaluation

Prepping the medical provider along with provision of a structured check list before embarking on a medical humanitarian aid mission (especially in short time frames such as in response to natural disasters) should be a vital and mandatory part of the mission planning phase in the homeland. Previous publications have shown that only 85% of NGOs briefed their field personnel before deployment to an acute emergency settings [11].

Mental implications related to participating in humanitarian aid delegations vary, and may include anxiety and depression which may occur before, during and after deployment. Burnout - a work-related syndrome- is also frequently encountered among mission personnel. These detrimental mental effects may be reduced in frequency and severity by implementing proper preparation measures [8, 11] such as reducing the uncertainty level by presenting relevant information during the briefings in the mission planning phase [7].

The IDF-MC takes great pride in its humanitarian aid efforts and emergency medicine response to natural disasters as do other military forces, and nowadays as in the past, these are still considered core military missions [12].Humanitarian response to crises employs an estimated 210000 people and accounts for billions of US dollars of spending annually [13]. One must also bear in mind that these undertakings are rarely considered effective [14] and are difficult to assess although various methods have been proposed [15]. Time allocated for preparation is scarce in most missions [16] and an optimal lengthy preparation process [17] is often not feasible. Thus, even in rapidly deployed missions, time allocated for personnel preparation and orientation may prove invaluable and ultimately may lead to a more efficient and effective execution phase.

## Conclusion

Preparation of medical personnel for humanitarian aid medical missions is a complex and vital task that might be better accomplished with thorough briefing and structured checklists which begin with addressing of personal safety and other daily needs of the staff. More research regarding how preparation affects mission success is needed as well as determining how to improve the preparation process itself. Preparation may raise performance levels, lessen stress and anxiety symptoms for the health care providers and ultimately assist in achieving the delegations’ goal of providing timely medical care to the local people struck by disaster.
